# Epidemiology of Alcohol Misuse and Illicit Drug Use Among Young People Aged 15–24 Years in Fishing Communities in Uganda

**DOI:** 10.3390/ijerph17072401

**Published:** 2020-04-01

**Authors:** Monica O. Kuteesa, Helen A. Weiss, Sarah Cook, Janet Seeley, Josephine N. Ssentongo, Robert Kizindo, Paul Ngonzi, Moses Sewankambo, Emily L. Webb

**Affiliations:** 1 MRC/UVRI and LSHTM Uganda Research Unit, Entebbe 49, Uganda; janet.seeley@lshtm.ac.uk (J.S.); Josephine.Naluwugge@mrcuganda.org (J.N.S.); robert.kizindo@mrcuganda.org (R.K.); pngonzi@gmail.com (P.N.); moses.sewankambo@mrcuganda.org (M.S.); 2 Department of Infectious Disease Epidemiology, London School of Hygiene and Tropical Medicine, Keppel Street, London WC 1E, UK; 3 Medical Research Council Tropical Epidemiology Group, London School of Hygiene and Tropical Medicine, London WC 1E, UK; helen.weiss@lshtm.ac.uk (H.A.W.);; 4 Department of Non-Communicable Disease Epidemiology, London School of Hygiene and Tropical Medicine, London WC 1E, UK; sarah.cook@lshtm.ac.uk; 5 Department of Global Health and Development, London School of Hygiene and Tropical Medicine, London WC 1E, UK

**Keywords:** alcohol, drugs, substance use, fisherfolk, adolescents, survey, Sub-Saharan Africa

## Abstract

Background: We determined the prevalence of and risk factors for alcohol misuse and illicit drug use among young Ugandans in fishing communities, a recognised “key population” for human immunodeficiency virus (HIV) infection. Methods: We conducted a cross-sectional survey among young people (15–24 years) in fishing communities in Koome, Uganda, in December 2017–July 2018. Using Audio-Assisted Self-Interviewing, we collected data on socio-demographic characteristics and alcohol use, including the Alcohol Use Disorders Identification Test (AUDIT) and timeline follow-back calendar (TLFB). Blood samples were analysed for HIV, herpes simplex virus 2 (HSV2), and Phosphatidyl ethanol (PEth 16:0/18:1). Urine samples were analysed for illicit drugs. Results: Among 1281 participants (52.7% male, mean age 20 years), 659 (51.4%) reported ever drinking alcohol, 248 (19.4%) had 12-month-AUDIT ≥ 8, and 261 (20.5%) had whole-blood PEth 16:0/18:1 concentration ≥ 20 ng/mL, indicating significant consumption. In multivariable analyses, PEth 16:0/18:1 ≥ 20ng/mL, AUDIT ≥ 8 and binge drinking (≥6 standard drinks per drinking occasion in the previous month from TLFB) were all strongly associated with older age, low education, smoking, and HSV2. Illicit drug use prevalence was 5.2% and was associated with older age, low education, being single, and smoking. Conclusion: Levels of alcohol misuse were high among young people in fishing communities and associated with HSV2, a proxy for risky sexual behaviour. Alcohol and illicit drug harm reduction services and HIV prevention programs in Uganda should prioritise young fisherfolk.

## 1. Introduction

Alcohol and illicit drug use are major global risk factors for disability and premature mortality and contribute to both communicable and non-communicable diseases [1,2]. This health burden is accompanied by significant economic costs, including expenditure on health care and law enforcement, loss of productivity and other direct and indirect costs including harm to others [1,2]. Adolescence is a vulnerability window for initiation and continuation of polysubstance use and associated harms [3]. For instance, globally, the prevalence of current drinking increases from 26.5% among 15–19 year olds to 40.7% among 20–24 year olds, while the prevalence of heavy episodic drinking increases from 13.6% to 21.8% [1]. An estimated 14% of all deaths among 20–39 year olds are attributable to alcohol [4].

The Sub-Saharan Africa (SSA) region has the highest estimated prevalence of heavy episodic drinking per drinker globally (25%), high levels of alcohol related disease and injury, large amounts of unrecorded consumption of illegally produced alcohol [1], and a growing market for alcohol sales [5]. An estimated 5.6% of young people aged 15–19 years (13.8 million) used cannabis in 2015 [4] and there are increasing reports of opioid and injection drug use [6]. Concurrent with the apparent increase in substance use in SSA, human immunodeficiency virus (HIV) remains a major public health problem, with young women aged 15–24 years and people who inject drugs disproportionately affected [7]. In a recent systematic review, we showed that in SSA, information on the burden of alcohol misuse and illicit drug use is limited, especially among young people belonging to certain occupational groups (i.e., sex workers, miners, truckers, fishing communities, uniformed personnel and motorcycle taxi riders) known to be at increased risk of HIV acquisition [8,9,10,11,12,13].

Fishing communities are a recognised key population group in Uganda, and some evidence suggests that as well as being at increased risk of HIV acquisition [10,14], they may also be at increased risk for substance use due to a combination of factors, including occupational norms, high mobility [15,16], geographical remoteness and inadequate substance use regulation [17,18]. However, studies on the burden of alcohol misuse remain few and diagnosis is challenging, as most studies have not used locally validated tools. The aim of this paper is to estimate the prevalence and correlates of alcohol misuse and illicit drug use, respectively, and their association with HIV risk behaviour, among young people in fishing communities on Lake Victoria in Uganda.

## 2. Materials and Methods

### 2.1. Study Setting, Design and Procedures

Between December 2017 and July 2018, we conducted a cross-sectional survey in fishing communities on the Koome Islands in Mukono District, Uganda, with an estimated population of 16,000. HIV prevalence in Koome was around 21% in adults aged 18+ in 2016 [19]. Based on data from a household survey conducted in 2013, as part of the LaVIISWA study [20], within the 15–25 year age group, reported alcohol misuse (defined as consuming at least five alcoholic drinks per day in the last 12 months on average), prevalence was 5%–6%.

The target population was young people aged 15–24 years and the primary outcome was prevalence of alcohol misuse. The target sample size of 1204 young people was calculated to allow us a precision of 2.5% with which to estimate a prevalence of alcohol misuse of up to 15% [19,21], assuming a design effect of 1.5. If the design effect was higher, at 2, then this sample size would allow us precision of 2.5% with which to estimate a prevalence of up to 11%. The target sample size would also provide 80% power to detect a difference in HIV/herpes simplex virus 2 (HSV2) prevalence of 20% versus 8% in young people who misuse alcohol compared to those who do not, assuming that prevalence of alcohol misuse is 12.5%, and the design effect is 2.

We used a two-stage sampling strategy, yielding a self-weighted sample. First, 20 villages were selected from the 27 villages on the islands using simple random sampling. Pre-existing village lists of households (defined as people who sleep in the same house and share meals) were available, but there was no list of individuals to use as a sampling frame. Therefore, we used the households as our sampling unit. We updated the village’s lists of households, and then sampled households from each selected village. Each household had a probability of 0.69 of selection; this probability was calculated to achieve the required sample size based on prior knowledge of the approximate number of young people per household. This approach ensured that all households across the study setting had equal probability of selection. We further assumed that the number of young people per household was consistent across the study setting. We included all young people in the selected households in our survey. This approach yielded a self-weighted sample. Young people aged 15–24 years and resident in the selected households were invited to participate and were enrolled after providing written informed consent. Non-emancipated minors gave assent and their parents/guardians provided parental consent. We excluded severely ill persons.

### 2.2. Data Collection

Survey data were collected by five trained interviewers (three psychiatry clinicians and two experienced non-clinical interviewers), privately, at the community hub or another agreed location. A questionnaire including items on socio-demographic characteristics of study participants, alcohol, tobacco and illicit drug use, depression, and sexual behaviour was developed. It was translated into the local language and back-translated into English separately by two trained translators; versions were compared, and necessary adjustments made to ensure accuracy. Most young people in Koome speak Luganda or English and the ability to do so was an inclusion criterion. All questions were then reviewed by community groups of adolescents from our target population for conceptual understanding and translation accuracy.

The questionnaire was administered using Audio Computer Assisted Self-Interviewing (ACASI). Respondents completed the questionnaire using a tablet and headphones, reading on their own and hearing a recorded voice speak the questions and response options. To build ACASI, both the English and Luganda versions of the questionnaire were audio recorded and programmed into the tablet by the research team, with skip patterns for questions that were not relevant. The questionnaire was pilot tested on 30 young people in a separate village in the same study area. Data collected from the pilot village were not included in this analysis; the results of the pilot were only used to modify survey collection instruments, field procedures, and data management systems, as necessary. During data collection, participants were provided with brief training on how to use the tablets and research staff were stationed nearby to answer questions and provide assistance, as necessary. Study participants were able to complete the interview in the language of their choice.

### 2.3. Measures

Demographic measures included age, religion, education, tribe, village, occupation and parity. Harmful alcohol use was assessed using the following measures. (i) The Alcohol Use Disorders Identification Test (AUDIT)—a 10-item screening tool developed by the World Health Organisation (WHO) to screen for harmful or hazardous drinking. An AUDIT score of ≥ 8 is considered to indicate hazardous or harmful alcohol use. The AUDIT has been validated across genders and in a wide range of racial/ethnic groups and is well-suited for use in primary care settings [22]. (ii) The timeline follow-back (TLFB) tool—a calendar method that can be used as a clinical and research tool to obtain a variety of quantitative estimates of alcohol or illicit drug use patterns over a specified time period that can vary from daily use up to 12 months from the interview date [23]. (iii) Phosphatidyl ethanol (PEth), a more sensitive and specific biomarker compared to traditional biomarkers (gamma-glutamyl transpeptidase (GGT), mean corpuscular volume (MCV) and carbohydrate-deficient transferrin (CDT)), for detecting chronic heavy drinking. PEth is detectable in whole blood for more than two weeks [24], and PEth ≥ 10 ng/mL has been shown to be highly sensitive (88%) and specific (89%) for any alcohol consumption among Ugandan adults living with HIV, in the prior 21 days [25]. Both AUDIT and TLFB tools were administered using ACASI while PEth was determined in dry blood spot (DBS) samples. Alcohol misuse based on AUDIT was defined as a score ≥ 8. Binge drinking based on TLFB was defined as ≥ 6 standard drinks per drinking occasion in the previous month. For this study, illicit drug use is defined as use of a substance where consumption has been prohibited by international drug control treaties except for medical purposes [26], and was assessed using urine tests. We also collected self-reported data on illicit drug use using the Alcohol, Smoking and Substance Involvement Screening Test (ASSIST), a screening tool recommended by the WHO to detect and manage substance use and related problems in primary and general medical care settings; it has been found to be feasible and have adequate test–retest reliability among adult populations in several geographic locations [27,28,29]. Depression severity was measured using the Patient Health Questionnaire (PHQ-9) [30]. Sexual risk behaviour was assessed by HSV2 and HIV results.

### 2.4. Laboratory Testing

To measure PEth, we used ethanol-free swabs for disinfection of the venous puncture site and collected capillary DBS samples. DBSs were dried at room temperature, without any direct sun, for three hours and stored in an airtight mini-grip bag with a drying agent in a fridge at <8 °C for up to 4 days. DBSs were then transported to the MRC/UVRI and LSHTM Uganda Research Unit reference laboratory in Entebbe and stored at −20 °C prior to express delivery on dry ice to the University of Bern, Switzerland, for analysis. DBSs were kept at −20 °C in the Bern laboratory and analysed within five to seven days of arrival to prevent in-vitro formation of PEth. Assays of PEth 16:0/18:1 and 16:0/18:2, the main PEth homologues in human blood [31] were conducted using high-performance liquid chromatography–tandem mass spectrometry (LC–MS/MS), using selected ion monitoring (SIM) in the negative mode of the deprotonated molecules. Here we report on PEth 16:0/18:1. Cut-offs for evaluating PEth 16:0/18:1 values were light or no consumption (< 20 ng/mL), significant consumption (20–209 ng/mL) and heavy consumption (≥ 210ng/mL) [32].

Additional laboratory tests included using a pre-defined nationally approved standard serial testing algorithm for HIV [33]: Alere Determine HIV 1/2 whole blood assay (Alere medical, Chiba, Japan) for screening followed by STAT-PAK rapid test HIV 1/2 (Chembio diagnostic systems New York, NY, USA) as confirmatory test and SD Bioline (Standard Diagnostics, Inc., Kyonggi-do, South Korea) as the tie breaker. For discordant results, confirmatory tests were conducted in an accredited laboratory using ELISA HIV (Murex, Diasorin: Ref 9E25-02, Saluggia VC, Italy) and Bio Elisa HIV1/2 Ag/Ab (BIOKIT, Barcelona, Spain). Newly diagnosed participants were referred for antiretroviral therapy initiation. HSV2 testing was performed at the Entebbe research laboratory using the Kalon ELISA (Kalon Biological, UK) with a cut-off optical density of 1.5. Fresh urine samples collected from all participants were tested at the study site for illicit drugs using a the (one-step rapid test, Protzek (Lörrach, Baden-Württemberg, Germany). This multiplate test is a sensitive immunochromatographic test based on a specific antigen–antibody reaction for the qualitative detection of amphetamines, methamphetamines, cocaine, ecstasy, opiates, and cannabinoids in human urine.

### 2.5. Data Management and Statistical Analysis

**Figure 1:**
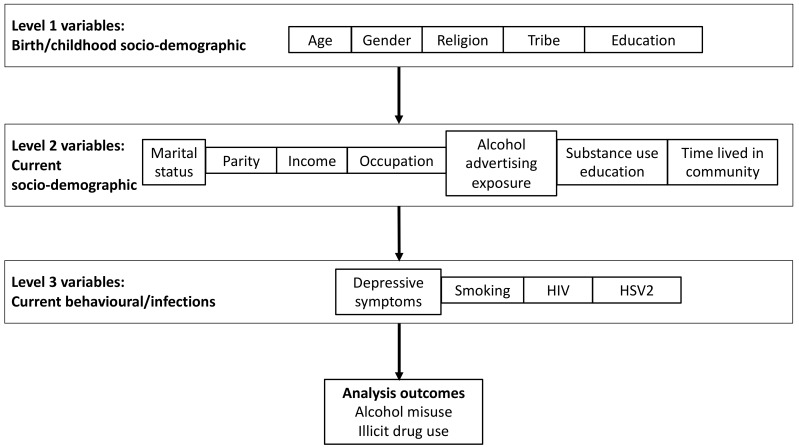
Conceptual framework for determinants of alcohol misuse and illicit drug use. HIV-Human Immunodeficiency Virus, HSV2-herpes Simplex Virus.

Data were captured electronically using an open data kit (ODK) and checked for missing fields and erroneous inputs. All statistical analyses were conducted using Stata, version 15 (Stata Corp, College Station, Texas, USA). We summarised participants’ socio-demographic characteristics and conducted univariable and multivariable analyses to identify factors associated with alcohol misuse (four separate outcomes: AUDIT ≥ 8, PEth ≥ 20ng/mL, PEth ≥ 210ng/mL, binge drinking based on TLFB) and illicit drug use. Multivariable analysis to control for potential confounding was guided by a hierarchical conceptual framework (Figure 1), following the approach described by Victora et al. [34]. We classified each variable into one of three groups (levels) from most distal to most proximal to the outcomes of interest for this analysis (alcohol misuse and illicit drug use). Level 1 variables were those determined at birth or during childhood, level 2 variables comprised current socio-demographic variables, and level 3 variables comprised current behaviour factors and infections. Level 1 variables were thought likely to impact on the outcomes through their effects on level 2 and hence on level 3 variables. Therefore, when assessing the effect of level 1 variables on the outcomes, we did not adjust for level 2 or level 3 variables in order to avoid adjusting for factors on the causal pathway. Similarly, when assessing the effect of level 2 variables on the outcomes, we did not adjust for level 3 variables. In summary, variables at the first level were adjusted for each other, variables at the second level were adjusted for each other and for first level variables, and third level variables were adjusted for each other and for first and second level variables. The only exception to this was that results for HIV were not adjusted for HSV2 and vice versa, since the two were both considered proxies for sexual behaviour and are highly correlated. Variables were retained in models regardless of their p-value after adjustment following this strategy; they were not excluded on the basis of high p-values. This strategy allowed us to assess the effects of variables at each level of the conceptual framework, having adjusted for more distal variables [34]. We used a two-stage sampling strategy and the sample was self-weighted. We accounted for clustering at the village and household level: logistic regression with a robust variance implemented using the svyset command in Stata was used to assess risk factors for alcohol misuse and illicit drug use.

### 2.6. Ethics Statement

We obtained ethical approval from the Uganda Virus Research Institute research and ethics committee (Ref: GC/127/17/07/595, approved on 27th July 2012), the London School of Hygiene and Tropical Medicine ethics committee (Ref: 14299, approved on 3rd October 2017), and the Uganda National Council for Science and Technology (Ref: SS 4385, approved on 6th October 2017) and the office of the president (Ref: ADM 194/212/01, approved on 20th October 2017). Any participants requiring medical treatment were referred to nearby government health centres where free treatment was offered.

## 3. Results

A total of 4521 households in the 20 villages were randomly selected for inclusion in the survey. Of these, 1115 were occupied by at least one young person aged 15–24 years. Residents of 146 households (representing 340 individuals) did not take part in the study, with not being located during the survey period (*n* = 144) and refusals (*n* = 196) being the most common reason for non-participation. A total of 1281 eligible young people from 969 households participated in the survey. Administration of ACASI questionnaires was acceptable and feasible; only two participants had difficulty completing the ACASI and were interviewed face-to-face. The final sample comprised 1281 young people aged 15–24 years (52.7% male; mean age 20 years (SD 2.7). The majority (61.3%) had attained primary education or less; 557 (43.5%) were single, with more females (253, 41.8%) reporting being married than males (158, 23.4%). Approximately 43% of the sample had lived in the fishing community for less than six months. Sixty-seven participants (5.2%) were living with HIV (1.8% male, 9.1% female) and 406 (32.0%) tested HSV2 positive (20.4% male, 44.9% female) (Table 1: participant characteristics). Missing values include participants who did not provide blood samples (*n* = 7); for the very small number of other missing results (HIV *n* = 2, HSV2 *n* = 4, PEth *n* = 5), the lack of results was a laboratory-based issue.

Overall, 51.4% of participants reported ever using any alcohol. The overall prevalence of alcohol misuse (AUDIT ≥ 8) was (19.4%, 95% CI:17.3-21.7), with males reporting this slightly more frequently (21.5% in males vs. 17.0% in females, *p* = 0.09, Table 1. A total of 73 (5.7%, 95% CI:4.5-7.2) participants reported an AUDIT score of ≥ 20 (classified as alcohol dependence; 8.0% in males and 3.1% in females). Among all participants, 261 (20.5%, 95% CI:18.2–22.7) had whole blood PEth 16:0/18:1 concentrations of ≥ 20ng/mL indicating significant drinking (24.6% in males vs. 15.9% in females, *p* < 0.001), 55 (4.3%, 95% CI:3.3–5.6) had whole blood PEth 16:0/18:1 concentrations of ≥ 210ng/mL, indicating heavy chronic drinking (5.5% in males vs. 3.0% in females, *p* = 0.03), and 81 (6.3%, 95% CI: 5.1-7.8) were classified as binge drinkers in the past month based on TLFB (8.9% male, 3.5% female, *p* = 0.002). The overall prevalence of self-reported lifetime use of tobacco or illicit drugs was (18.4%, 95% CI: 16.3-20.6); 26.1% males vs. 9.7% females, and the overall prevalence of recent illicit drug use based on a urine test was 5.2% (7.7% males, 2.4% females, *p* = 0.02). Among participants with a positive urine drug test, the only drugs detected were cannabinoids (*n* = 58, 90.6%) and amphetamines (*n* = 6, 9.4%).

### Risk Factors for Substance Use

Table 2 shows the relationship between examined characteristics and alcohol misuse (AUDIT ≥ 8) in the study. In univariable analyses, the prevalence of alcohol misuse (AUDIT ≥ 8) was higher in those aged 20–24 years compared to 15–19-year olds (Odds-Ratio (OR) 1.89, 95% Confidence Interval (CI): 1.37-2.62). AUDIT ≥ 8 was not associated with gender (either in the full sample or when restricted only to those who reported any drinking), but was crudely associated with all other characteristics examined except marital status, duration of time spent in the fishing community, and whether information on the dangers of alcohol misuse were taught in school. After adjustment for potential confounders, AUDIT ≥ 8 remained strongly associated with older age, lower education, fishing-related occupation status, being non-Ugandan, smoking (aOR 4.45, 95% CI: 2.84–6.97), HSV2 (aOR 1.87, 95% CI: 1.34–2.59) and depressive symptoms (compared to minimal symptoms, aOR 2.35, 95% CI: 1.41-3.91 and aOR 2.30, 95% CI: 0.53–9.90 for mild and moderate-severe, respectively) (Table 2). After adjustment, HIV was no longer associated with AUDIT ≥ 8 (aOR 1.50, 95% CI: 0.72–3.13).

Table 3 shows the relationship between the examined characteristics and PEth 16:0/18:1 ≥ 20ng/mL (representing heavy drinking). In univariable analysis, all characteristics were associated with PEth 16:0/18:1 ≥ 20ng/mL except for duration of time lived in the community and exposure to alcohol adverts. In multivariable analyses, alcohol misuse according to PEth remained strongly associated with male gender, older age, low education, being non-Ugandan, being non-Muslim, occupations in itinerant trade, fish-related or entertainment industries, parity, smoking (aOR 3.16, 95% CI: 1.76–5.68), illicit drug use (aOR 2.72, 95% CI: 1.29–5.74) and HSV2 (aOR 1.87, 95% CI: 1.36–2.56). After adjustment, HIV was no longer associated with PEth 16:0/18:1 ≥20ng/mL (aOR 1.43, 95% CI: 0.93–2.19).

Supplementary Table S1 shows that factors associated with hazardous drinking (PEth 16:0/18:1 ≥210ng/mL) at multivariable analysis included male gender, older age group 20–24 and low education.

Table 4 shows the relationship between the characteristics and binge drinking in the past month ascertained using TLFB. In crude analysis, binge drinking was associated with all characteristics except tribe, occupation, duration in community, exposure to school curriculum on alcohol, and depressive symptoms. After adjusting for potential confounders, binge drinking was associated with older age, low education, being single, parity, exposure to alcohol adverts, and smoking, illicit drug use and HSV2, with weak evidence for association with mild depressive symptoms.

Table 5 shows the relationship between the examined characteristics and illicit drug use (positive urine test) in adolescents. In univariable analysis, the prevalence of illicit drug use was associated with gender, age, education, income, occupation, smoking and depressive symptoms. After adjustment for potential confounders, illicit drug use remained positively associated with male gender, older age group, lower education, being single, smoking, and moderate to severe depressive symptoms.

## 4. Discussion

Alcohol misuse was common among young people aged 15–24 years in fishing communities in Uganda, and was consistently associated with older age, male gender (except for AUDIT ≥ 8), lower education, smoking, HSV2 and depressive symptoms, regardless of the alcohol use assessment tool used. The prevalence of illicit drug use was lower than that of alcohol misuse. The only drugs detected were cannabinoids and amphetamines. Illicit drug use was associated with male gender, older age, lower education, being single, smoking, and depressive symptoms.

There are limited data on alcohol misuse and illicit drug use from similar settings [8]. The prevalence of alcohol misuse in our study was comparable to findings in a recent study among young people in a general population setting in Northern Tanzania, with 11%–28% of males screening positive for alcohol use disorder (AUD) [35], and findings among young people in Goa, India (17.7%) [36]. However, the prevalence of AUD in our study was much lower than the prevalence (53%, measured by CAGE) among 14–24 year old female sex workers in Kampala [37], as well as in another study of a key population where, among 1476 young men who reported sex with men in Kenya, 44% had reported AUD via the AUDIT questionnaire [38]. The prevalence of binge drinking in our study was slightly lower than that reported among young people in the WHO Africa region comprising 47 countries [1]. Young men were more likely to screen positive for both AUD based on PEth and binge drinking, and this is consistent with estimates from Africa and other WHO regions [1], although interestingly, AUDIT ≥ 8 was not associated with gender in this setting. Taken together, these studies further highlight the need for integration and prioritisation of alcohol reduction in HIV prevention interventions among key populations.

The prevalence of illicit drug use was 4.4%, similar to a large population-based survey in South Africa among people aged 15 and older [39]. The most commonly detected drug was cannabis, and this might be attributed to better availability and social acceptability, or lower cost compared to other illicit drugs.

Globally, predictors of harmful alcohol use and illicit drug use among young people include male gender, peer pressure, family experience, psychological factors/mental health, positive expectations regarding alcohol use, violence, socio-economic factors, and increased alcohol and illicit drug availability and marketing [35,40,41]. Furthermore, young people are potentially more likely to engage in reckless behaviour while drunk [42] or whilst on drugs than older adults. In our study, socio-demographic characteristics associated with AUD were similar to those found in studies conducted in more general population settings elsewhere in East Africa and Europe/US. They included male gender, older age group (20–24 years), using illicit drugs and HSV2 infection [37]. However, other studies among female sex workers reported contrary risk factors such as younger age [37]. The findings emphasize the need for integration of alcohol and illicit drug use prevention interventions, and for interventions that will be able to reach males.

Extensive evidence shows that alcohol misuse and illicit drug use are associated with higher HIV risk at societal, community and individual levels^,^ stemming mainly from associations with high risk sexual behaviour, increased HIV shedding and inflammation at mucosal sites [43] and needle sharing for injection drug users [44]. Among people living with HIV, alcohol and illicit drug use may adversely affect disease progression through several mechanisms, including reduced uptake of HIV prevention and care services [45], and through impacts on viral replication, host immunity and treatment compliance and efficacy [43]. Our cross-sectional study did not show a significant association between HIV and AUD or illicit drug use after adjustment for potential confounders, although adjusted odds ratios were greater than 1 for all measures of AUD, and lower limits of 95% confidence intervals only marginally overlapped 1. The lack of significant relationships between these variables may be in part due to the relatively small number of HIV events and consequent limited power to detect an association. However, they should not be interpreted as evidence for the absence of such relationships. There was a strong association between substance use and HSV2. Evidence suggests a direct and reciprocal biological interaction between HIV and HSV2 [46]. Additionally, HIV and HSV2 are associated with similar risk factors [age, sex, partner change, condom use and male circumcision) [46]. Thus, HSV2 may be considered a proxy measure for risky sexual behaviour. Therefore, prioritizing alcohol interventions and harm reduction among young people in key population settings is critical not only to reducing substance-use-related harms but also to reducing sexually transmitted infections and could accelerate progress towards the UNAIDS 95-95-95 HIV epidemic control targets. Our study documents both under-age alcohol use and misuse (the legal age for drinking in Uganda is 18). This implies that additional research to understand the drivers of substance use among minors and interventions to address them is critical.

### Strengths and Limitations

A strength of the study was the large sample size, which meant that we had good power to detect associations with alcohol misuse for most covariates of interest. We selected a population-based sample and the response rate was high; thus, our findings may be generalisable to similar fishing community settings in the Eastern and Southern Africa regions, although we did not collect detailed information on those who did not participate to further assess this. Another strength is that we used a range of standard tools to assess alcohol use, encompassing the different types of alcohol consumption measures, i.e., tools that report alcohol consumption status, average volume alcohol consumption, and frequency and volume of binge drinking. We identified several key covariates that were strongly associated with alcohol use across all three measures used. These standard tools are essential for monitoring public health and evaluating alcohol control policies and other interventions.

Our results might be less prone to social desirability bias/misreporting, which could bias our estimates of association either towards or away from the null, for two reasons: (i) use of objective measures to supplement self-reported measures of alcohol and illicit drug use and sexual risk behaviour; (ii) administration of ACASI, which was acceptable and feasible. Previous literature suggests that ACASI allows more accurate reporting of sensitive behaviours including alcohol (mis)use. However, we did not have an interviewer-administered comparison group to test this hypothesis in our study. Therefore, our findings might not be generalizable to AUDIT being administered face-to-face. We further minimised reporting bias by using young gender-matched interviewers, and by using a customised TLFB calendar and pictorial displays of standard alcoholic drinks [35]. Our survey had a response rate (based on residents listed in a previous census) of approximately 80%, therefore we cannot exclude the possibility that our results are subject to selection bias. Non-response, especially in a study of a controversial topic such as substance abuse or sexual behaviour, could potentially introduce selection bias and consequently lead to underestimation of outcome and risk factor prevalence [47], and may affect the internal validity of the study [48]. Interpretation of the effect sizes found in this study should be undertaken with caution, as these may depend on the specific characteristics of the samples included in our study. A further limitation of our study was the cross-sectional design, which precludes us from demonstrating causality between independent variables and AUD and or illicit drug use, and between AUD and HIV or HSV2. We chose to model HIV and HSV2 as risk factors for alcohol misuse and illicit drug use since alcohol misuse and illicit drug use were our primary outcomes of interest, but reverse causation is a plausible explanation for the associations seen.

## 5. Conclusions

Alcohol misuse and illicit drug use are common among young people in fishing communities in Uganda and associated with sexual risk behaviour and smoking. Uganda is an HIV-hyperendemic country, with one of the youngest and fastest-growing populations and one of the highest estimated alcohol per capita consumption rates globally [1]. There is potential for additional benefit of combined prevention and intervention activities targeting alcohol and illicit drug use, and HIV particularly, in key population settings.

## Figures and Tables

**Table 1:** Participant characteristics.

Characteristics	Categories	Men (*n* = 675)		Women (*n* = 606)		Overall (*n* = 1281)	
		n	%	N	%	N	%
Age	15–19 years	261	38.7	226	37.3	487	38.0
	20–24 years	414	61.3	380	62.7	794	62.0
Religion	Christian	505	74.8	461	76.1	966	75.4
	Muslim	156	23.1	145	23.9	301	23.5
	Traditional/other	14	2.1	0	0	14	1.1
Marital status	Married	158	23.4	253	41.8	411	32.1
	Cohabiting	106	15.7	118	19.5	224	17.5
	Single	378	56.0	180	29.7	558	43.6
	Separated/divorced/widowed	33	4.9	55	9.1	88	6.9
Education attainment	None	22	3.3	15	2.5	37	2.9
	Incomplete primary	338	50.1	262	43.2	600	46.8
	Complete primary	76	11.3	73	12.1	149	11.6
	Secondary junior	210	31.1	230	38	440	34.4
	Secondary senior and above	29	4.3	26	4.3	55	4.3
Time resident in fishing community	< 6 months	254	37.6	296	48.8	550	42.9
	7 to 11 months	114	16.9	100	16.5	214	16.7
	1 to 5 years	221	32.7	170	28.1	391	30.5
	6 to 10 years	37	5.5	16	2.6	53	4.1
	>11 years	49	7.3	24	4	73	5.7
							
Human Immunodeficiency Virus (HIV *)	Positive	12	1.8	55	9.1	67	5.3
	Negative	657	98.2	548	90.9	1205	94.7
							
Herpes Simplex Virus (HSV2 **)	Positive	136	20.4	270	44.9	406	32.0
	Negative	532	79.6	332	55.2	864	68.0
							
Ever used alcohol	Yes	363	53.8	296	48.8	659	51.4
	No	312	46.2	310	51.2	622	48.6
							
30-day Alcohol Use Disorders Identification Test (AUDIT)	Mean (SD)	5.5	8.0	3.7	6.3	4.7	7.3
	<7 Low risk or non-drinker	480	71.1	492	81.2	972	75.9
	8-15 Excess of low risk	105	15.6	70	11.6	175	13.7
	16-19 Harmful drinking	28	4.2	18	3.0	46	3.6
	≥20 Alcohol dependence	62	9.2	26	4.3	88	6.9
							
12-month AUDIT	Mean (SD)	4.7	7.7	3.3	6.2	4.0	7.0
	<7 low risk or non-drinker	530	78.5	503	83	1033	80.6
	8–15 Excess of low risk	72	10.7	60	9.9	132	10.3
	16–19 Harmful drinking	19	2.8	24	4	43	3.4
	≥20 Alcohol dependence	54	8	19	3.1	73	5.7
							
Phosphatidylethanol (Peth) cut-off for alcohol use (PEth 16:0/18:1) ***	Light or no consumption	505	75.4	510	84.2	1015	79.5
	Heavy drinking (20–209 ng/mL)	128	19.1	78	12.9	206	16.1
	Hazardous drinking (≥210 ng/mL)	37	5.5	18	3.0	55	4.3
							
Timeline Followback(TLFB) (≥6 standard drinks per drinking occasion, in previous month)							
	No	615	91.1	585	96.5	1200	93.7
	Yes	60	8.9	21	3.5	81	6.3
							
Urine drug test	Positive	50	7.7	14	2.4	64	5.2
	Negative	600	92.3	575	97.6	1175	94.8
							
Ever used tobacco or illicit drugs ****	No	499	73.9	547	90.3	1046	81.7
	Yes	176	26.1	59	9.7	235	18.4

*Missing HIV status for 6 men, 3 women; **missing HSV2 status for 7 men, 4 women; *** missing PEth for 5 men; **** in the study community, tobacco is often smoked with a mixture of other illicit drugs. Prevalence of substance use. HIV-Human Immunodeficiency Virus, HSV2-herpes Simplex Virus. AUDIT- Alcohol Use Disorders Identification Test. SD – standard deviation.

**Table 2:** Risk factors for alcohol misuse (AUDIT score ≥8) among young people aged 15–24 years in fishing communities in Uganda.

Characteristic	Categories	Low Drinking (AUDIT ≤7)	Heavy Drinking (AUDIT ≥8)	Crude Odds Ratio (95% CI)	*p*-Value	Adjusted Odds Ratio (95%CI) *	*p*-Value
	All participants	1281 (80.6)	248 (19.4)				
							
Gender	Male	530 (78.5)	145 (21.5)	1 (Reference)		1 (Reference)	
	Female	503 (83.0)	103 (17.0)	0.75 (0.54–1.05)	0.09	0.80 (0.57–1.14)	0.20
							
Age group	15-19	421 (86.5)	66 (13.6)	1 (Reference)		1 (Reference)	
	20-24	612 (77.1)	182 (22.9)	1.89 (1.37–2.62)	0.001	1.97 (1.46–2.67)	<0.001
							
Education	None/primary	605 (77.0)	181 (23.0)	1 (Reference)		1 (Reference)	
	Secondary and above	428 (86.5)	67 (13.5)	0.52 (0.34–0.799)	0.005	0.49 (0.33-0.74)	0.002
							
Tribe	Central	390 (81.4)	89 (18.6)	1 (Reference)		1 (Reference)	
	Eastern	356 (82.8)	74 (17.2)	0.91 (0.64–1.30)		0.87 (0.62–1.21)	
	Northern	27 (90.0)	3 (10.0)	0.49 (0.14–1.73)		0.48 (0.15–1.50)	
	South-Western	164 (81.6)	37 (18.4)	0.99 (0.65–1.51)		0.86 (0.56–1.32)	
	Non-Ugandan	77 (64.7)	42 (35.3)	2.39 (1.62–3.52)		2.22 (1.48–3.32)	
	Other	19 (86.4)	42 (35.3)	0.69 (0.218–2.2)	0.006	0.60 (0.18–2.00)	0.01
							
Religion	Christian	767 (79.4)	199 (20.6)	1 (Reference)		1 (Reference)	
	Muslim	257 (85.4)	44 (14.6)	0.66 (0.43–1.03)		0.68 (0.43–1.06)	
	Traditional/other	9 (64.3)	5 (35.71)	2.14 (0.85–5.38)	0.04	1.82 (0.64–5.17)	0.10
							
Parity	0	574 (83.7)	112 (16.3)	1 (Reference)		1 (Reference)	
	1	258 (74.6)	88 (25.4)	1.75 (1.20–2.55)		1.65 (1.05–2.60)	
	≥2	201 (80.7)	48 (19.3)	1.22 (0.82–1.83)	0.02	1.22 (0.73–2.04)	0.08
							
Marital status	Cohabiting	171 (76.3)	53 (23.7)	1 (Reference)		1 (Reference)	
	Married	340 (82.7)	71 (17.3)	0.67 (0.45–1.02)		0.57 (0.38–0.87)	
	Separated/divorced/widowed	65 (73.9)	23 (26.1)	1.14 (0.73–1.79)		0.83 (0.47–1.47)	
	Single	457 (81.9)	101 (18.1)	0.71 (0.48–1.07)	0.15	0.89 (0.57–1.40)	0.10
							
Income	<200	642 (83.6)	126 (16.4)	1 (Reference)		1 (Reference)	
	200 K-299 K	162 (76.1)	51 (23.9)	1.60 (1.11-2.32)		1.05 (0.71–1.57)	
	300 K-399 K	121 (81.2)	28 (18.8)	1.17 (0.75-1.86)		0.85 (0.45–1.58)	
	≥400 K	108 (71.5)	43 (28.5)	2.03 (1.36-3.03)	0.002	1.35 (0.77–2.36)	0.59
							
Occupation	Agriculture	167 (82.7)	35 (17.3)	1 (Reference)		1 (Reference)	
	Entertainment	148 (75.1)	49 (24.9)	1.58 (0.95-2.63)		1.41 (0.82–2.41)	
	Fishing related	268 (70.2)	114 (29.8)	2.03 (1.21–3.41)		2.05 (1.18–3.30)	
	Itinerary trade	164 (87.7)	23 (12.3)	0.67 (0.36–1.25)		0.67 (0.37–1.23)	
	Unemployed	250 (91.2)	24 (8.8)	0.46 (0.27–0.77)		0.53 (0.30–0.90)	
	Other	36 (92.3)	3 (7.7)	0.40 (0.06–2.73)	<0.001	0.50 (0.08–3.07)	<0.001
							
Duration in community	≤6 months	453 (82.4)	97 (17.64)	1 (Reference)		1 (Reference)	
	7–12 months	173 (80.8)	41 (19.2)	1.11 (0.79–1.56)		1.09 (0.72–1.63)	
	1–4 years	311 (79.5)	80 (20.5)	1.20 (0.84–1.71)		1.00 (0.68–1.47)	
	≥5 years	96 (76.2)	30 (23.8)	1.46 (0.89–2.39)	0.52	1.10 (0.64–1.87)	0.94
							
Alcohol adverts	No	227 (86.3)	36 (13.7)	1 (Reference)		1 (Reference)	
	Yes	806 (79.2)	212 (20.8)	1.66 (1.02–2.70)	0.04	1.62 (0.95–2.79)	0.08
							
School curriculum	No	333 (78.9)	89 (21.1)	1 (Reference)		1 (Reference)	
	Yes	700 (81.5)	159 (18.51)	0.85 (0.61–1.18)	0.31	1.02 (0.69–1.49)	0.94
							
Smoking	No	923 (86.0)	150 (14.0)	1 (Reference)		1 (Reference)	
	Yes	110 (52.9)	98 (47.1)	5.48 (3.90–7.70)	<0.001	4.45 (2.84–6.97)	<0.001
							
Illicit drug use	No	975 (82.4)	209 (17.7)	1 (Reference)		1 (Reference)	
	Yes	27 (49.1)	28 (50.9)	4.73 (3.11–7.20)	<0.001	1.38 (0.78–2.43)	0.25
							
HIV	Negative	978 (81.2)	227 (18.8)	1 (Reference)		1 (Reference)	
	Positive	47 (70.2)	20 (29.9)	4.83 (3.03–7.72)	<0.001	1.50 (0.72–3.13)	0.27
							
HSV2	Negative	728 (84.3)	136 (15.7)	1 (Reference)		1 (Reference)	
	Positive	295 (72.7)	111 (27.3)	2.01 (1.55–2.61)	<0.001	1.87 (1.34–2.59)	<0.001
							
Depressive symptoms	Minimal	931 (82.6)	196 (17.4)	1 (Reference)		1 (Reference)	
	Mild	82 (65.6)	43 (34.4)	2.49 (1.43–4.34)		2.35 (1.41–3.91)	
	Moderate–severe	15 (68.2)	7 (31.8)	2.22 (1.10–4.44)	0.01	2.30 (0.53–9.90)	0.01

*All adjusted ORs controlled for age, gender, education, tribe and religion; adjusted ORs for parity, marital status, income, occupation, duration in community, alcohol adverts and school curriculum additionally controlled for each other; adjusted ORs for smoking, alcohol use, depressive symptoms, HIV and HSV2 additionally adjusted for each other (with the exception of HIV and HSV2, which were not controlled for each other). CI-confidence interval.

**Table 3:** Risk factors for alcohol misuse (PEth 16:0/18:1 ≥20 ng/mL) among young people aged 15–24 years in fishing communities in Uganda.

Characteristic	Categories	Not Detected (PEth 16:0/18:1 <20 ng/mL) N(%)	Heavy Drinking (PEth 16:0/18:1 ≥20 ng/mL) N(%)	Crude Odds Ratio (95% CI)	*p*-Value	Adjusted Odds Ratio (95%CI)	*p*-Value
	All participants	1015 (79.5)	261 (20.5)				
							
Gender	Male	505 (75.4)	165 (24.6)	1 (Reference)		1 (Reference)	
	Female	510 (84.2)	96 (15.8)	0.58 (0.44–0.76)	<0.001	0.58 (0.42–0.81)	0.003
							
Age	15-19	440 (90.4)	47 (9.7)	1 (Reference)		1 (Reference)	
	20-24	575 (72.9)	214 (27.1)	3.48 (2.28–5.31)	<0.001	3.72 (2.45–5.63)	<0.001
							
Education	None/primary		189 (24.1)	1 (Reference)		1 (Reference)	
	Secondary and above	420 (85.4)	72 (14.6)	0.54 (0.40–0.72)	<0.001	0.48 (0.36-0.65)	<0.001
							
Tribe	Central	374 (78.7)	101 (21.3)	1 (Reference)		1 (Reference)	
	Eastern	358 (83.5)	71 (16.6)	0.73 (0.53–1.02)		0.70 (0.49–1.00)	
	Northern	27 (90.0)	3 (10.0)	0.41 (0.15–1.12)		0.42 (0.18–0.99)	
	South-Western	154 (76.6)	47 (23.4)	1.13 (0.68–1.89)		0.96 (0.57–1.62)	
	Non-Ugandan	82 (68.9)	37 (31.1)	1.67 (1.06–2.63)		1.50 (0.93–2.41)	
	Other	20 (90.9)	2 (9.1)	0.30 (0.09–1.49)	0.02	0.30 (0.07–1.37)	0.04
							
Religion	Christian	751 (78.1)	211 (21.9)	1 (Reference)		1 (Reference)	
	Muslim	256 (85.3)	44 (14.7)	0.61 (0.47-0.80)		0.63 (0.49–0.81)	
	Traditional/Other	8 (57.1)	6 (42.9)	2.27 (0.96-7.41)	0.001	2.05 (0.68–6.17)	0.01
							
Parity	0	571 (83.5)	113 (16.5)	1 (Reference)		1 (Reference)	
	1	249 (72.2)	96 (27.8)	1.95 (1.32–2.87)		1.70 (1.06–2.73)	
	≥2	195 (79)	52 (21.1)	1.35 (0.84–2.16)	0.008	1.22 (0.66–2.36)	0.04
							
Marital status	Cohabiting	170 (76.2)	53 (23.8)	1 (Reference)		1 (Reference)	
	Married	330 (80.9)	78 (19.1)	0.76 (0.53-1.09)		0.60 (0.40-0.92)	
	Separated/divorced/ widowed	60 (68.2)	28 (31.8)	1.50 (0.71-3.14)		1.02 (0.41–2.54)	
	Single	455 (81.7)	102 (18.3)	0.72 (0.53–0.97)	0.04	0.93 (0.60–1.47)	0.13
							
Income	<200	644 (84)	123 (16)	1 (Reference)		1 (Reference)	
	200–299 K	146 (68.9)	66 (31.1)	2.37 (1.63–3.44)		1.35 (0.81–2.25)	
	300–399 K	114 (77.0)	34 (23.0)	1.56 (1.07–2.27)		0.59 (0.60–1.51)	
	≥400 K	111 (74.5)	38 (25.5)	1.79 (1.15–2.79)	0.002	0.92 (0.51–1.67)	0.25
							
Occupation	Agriculture	176 (87.1)	26 (12.9)	1 (Reference)		1 (Reference)	
	Entertainment	152 (77.2)	45 (22.8)	2 (1.22–3.3)		2.06 (1.19–3.54)	
	Fishing related	250 (66.3)	127 (33.7)	3.34 (2.24–5.05)		3.01 (1.81–5.00)	
	Itinerary trade	150 (80.2)	37 (19.8)	1.67 (1.08–2.58)		1.90 (1.20–3.05)	
	Unemployed	252 (92.0)	22 (8.0)	0.59 (0.30–1.15)		0.94 (0.43–2.09)	
	Other	35 (89.7)	4 (10.3)	0.77 (0.22–2.71)	<0.001	0.93 (0.29–2.94)	0.004
							
Duration in community	≤6 months	444 (80.9)	105 (19.1)	1 (Reference)		1 (Reference)	
	7 months to 1 year	169 (79.3)	44 (20.7)	1.10 (0.77-1.57)		1.00 (0.67–1.47)	
	1–4 years	303 (78.1)	85 (21.9)	1.19 (0.93–1.52)		0.88 (0.61–1.26)	
	≥5 years	99 (78.6)	27 (21.4)	1.15 (0.68–2.00)	0.53	0.70 (0.38–1.30)	0.63
							
Alcohol adverts	No	224 (85.5)	38 (14.5)	1 (Reference)		1 (Reference)	
	Yes	791 (78.0)	223 (22.0)	1.66 (0.95–2.90)	0.07	1.69 (0.97–3.00)	0.07
School curriculum	No	318 (75.4)	104 (24.6)	1 (Reference)		1 (Reference)	
	Yes	697 (81.6)	157 (18.4)	0.69 (0.50–0.95)	0.02	0.76 (0.52–1.10)	0.14
							
Smoking	No	902 (84.4)	167 (15.6)	1 (Reference)		1 (Reference)	
	Yes	113 (54.6)	94 (45.4)	4.49 (3.19–6.33)	<0.001	3.16 (1.76-5.68)	<0.001
							
Illicit drug use	No	964 (81.6)	218 (18.4)	1 (Reference)		1 (Reference)	
	Yes	21 (38.2)	34 (61.8)	7.03 (4.25–11.64)	<0.001	2.72 (1.29–5.74)	0.01
							
HIV	Negative	968 (80.6)	233 (91.4)	1 (Reference)		1 (Reference)	
	Positive	45 (67.2)	22 (32.8)	2.03 (1.5-2.75)	<0.001	1.43 (0.93-2.19)	0.10
							
HSV2	Negative	721(83.6)	141 (16.4)	1 (Reference)		1 (Reference)	
	Positive	291(71.9)	114 (28.2)	2.22 (1.59-2.52)	<0.001	1.87 (1.36-2.56)	<0.001
							
Depressive symptoms	Minimal	907 (80.8)	216 (19.2)	1 (Reference)		1 (Reference)	
	Mild	86 (69.4)	38 (30.7)	1.85 (1.08-3.2)		1.75 (0.94-3.26)	
	Moderate–severe	22(75.9)	7 (24.1)	1.33 (0.74-2.4)	0.02	1.26 (0.45-3.50)	0.15

**Table 4:** Risk factors for alcohol misuse (binge drinking in previous month) among young people aged 15–24 years in fishing communities in Uganda.

Characteristics	Categories	Binge Drinking N (%)	Crude Odds Ratio (95% CI)	*p*-Value	Adjusted Odds Ratio (95%CI) *	*p*-Value
	All participants	81 (6.3)				
						
Gender	Male	60 (8.9)	1 (Reference)		1 (Reference)	
	Female	21 (3.5)	0.37 (0.20–0.67)	0.002	0.39 (0.22–0.70	0.03
						
Age group	15–19	22 (4.5)	1 (Reference)		1 (Reference)	
	20–24	59 (7.4)	1.70 (0.99–2.92)	0.06	1.77 (1.02–3.08)	0.05
						
Education	None/primary	62 (7.9)	1 (Reference)		1 (Reference)	
	Secondary and above	19 (3.8)	0.47 (0.22–1.00)	0.05	0.46 (0.21–0.97)	0.04
						
Tribe	Central	30 (6.3)	1 (Reference)		1 (Reference)	
	Eastern	18 (4.2)	0.65 (0.29–1.46)		0.63 (0.29–1.38)	
	Northern	2 (6.7)	1.07 (0.26–4.49)		1.12 (0.31–4.05)	
	South-Western	15 (7.5)	1.21 (0.50–2.91)		1.08 (0.46–2.54)	
	Non-Ugandan	14 (11.8)	2.00 (1.08–3.38)		1.69 (0.90–3.18)	
	Other	2 (9.1)	1.50 (0.33–0.12)	0.31	1.21 (0.27–5.41)	0.48
						
Religion	Christian	68 (7.0)	1 (Reference)		1 (Reference)	
	Muslim	11 (3.7)	0.50 (0.28–0.89)		0.55 (0.31–0.98)	
	Traditional/other	2 (14.3)	2.20 (0.50–9.73)	0.02	1.42 (0.34–6.02)	0.09
						
Parity	0	34 (5.0)	1 (Reference)		1 (Reference)	
	1	33 (9.5)	2.02 (1.28–3.19)		2.77 (1.59–4.83)	
	≥2	14 (5.6)	1.14 (0.56–2.32)	<0.001	2.02 (0.93–4.39)	0.00
						
Marital status	Cohabiting	15 (6.7)	1 (Reference)		1 (Reference)	
	Married	23 (5.6)	0.83 (0.49–1.40)		0.59 (0.32–1.09)	
	Separated/divorced/widowed	7 (8.0)	1.20 (0.31–4.63)		0.84 (0.24–2.87)	
	Single	36 (6.5)	0.96 (0.43–2.13)	0.43	1.10 (0.38–3.17)	0.01
						
Income	<200	34 (4.4)	1 (Reference)		1 (Reference)	
	200K–300K	23 (10.8)	2.61 (1.39–4.91)		1.59 (0.76–3.30)	
	300K–400K	11 (7.4)	1.72 (0.89–3.34)		1.12 (0.51–2.48)	
	≥400K	13 (8.6)	2.03 (1.05–3.93)	0.02	1.11 (0.51–2.42)	0.51
						
Occupation	Agriculture	12 (5.9)	1 (Reference)		1 (Reference)	
	Entertainment	7 (3.6)	0.58 (0.19–1.82)		0.66 (0.24–1.85)	
	Fishing related	45 (11.8)	2.11 (0.83–5.37)		1.60 (0.54–4.73)	
	Itinerary trade	5 (2.7)	0.44 (0.13–1.45)		0.50 (0.14–1.82)	
	Unemployed	8 (2.9)	0.48 (0.22–1.02)		0.74 (0.35–1.54)	
	Other	4 (10.3)	1.81 (0.41–7.94)	0.07	2.40 (0.49–11.71)	0.34
						
Duration in community	≤6 months	28 (5.1)	1 (Reference)		1 (Reference)	
	7 months to 1 year	10 (4.7)	0.91 (0.45–1.87)		0.71 (0.34–1.47)	
	1 -4 years	28 (7.2)	1.44 (0.88–2.35)		0.96 (0.56–1.63))	
	≥ 4 years	15 (11.9)	2.52 (1.28-4.95)	0.09	1.55 (0.73-3.28)	0.29
						
Alcohol adverts	No	9 (3.4)	1 (Reference)		1 (Reference)	
	Yes	72 (7.1)	2.15 (1.37–3.38)	0.002	2.09 (1.17–3.73)	0.02
						
School curriculum	No	31 (7.4)	1 (Reference)		1 (Reference)	
	Yes	50 (5.8)	0.78 (0.42–1.43)	0.40	0.90 (0.51–1.62)	0.72
						
Smoking	No	47 (4.4)	1 (Reference)		1 (Reference)	
	Yes	34 (16.4)	4.27 (2.52–7.22)	<0.001	2.34 (1.28–4.27)	0.01
						
Illicit drug use	No	59 (5.0)	1 (Reference)		1 (Reference)	
	Yes	17 (26.6)	6.84 (3.97–11.80)	<0.001	2.54 (1.00–6.41)	0.05
						
HIV	Negative	72 (6.0)	1 (Reference)		1 (Reference)	
	Positive	9 (13.4)	2.44 (1.06–5.64)	<0.001	2.97 (0.82–10.73)	0.09
						
HSV2	Negative	372 (31.3)	1 (Reference)		1 (Reference)	
	Positive	34 (42.0)	1.59 (0.97–2.60)	0.06	1.77 (1.03–3.04)	0.04
						
Depressive symptoms	Minimal	64 (5.7)	1 (Reference)		1 (Reference)	
	Mild	14 (11.2)	2.09 (1.13–3.89)		2.25 (1.14–4.45)	
	Moderate–severe	3 (10.3)	1.92 (0.60–6.09)	0.06	2.64 (0.28–25.40)	0.05

*All adjusted ORs controlled for age, gender, education, tribe and religion; adjusted ORs for parity, marital status, income, occupation, duration in community, alcohol adverts and school curriculum additionally controlled for each other; adjusted ORs for smoking, illicit drug use, depressive symptoms, HIV and HSV2 additionally adjusted for each other (with the exception of HIV and HSV2, which were not controlled for each other).

**Table 5:** Risk factors for illicit drug use among young people aged 15–24 years in fishing communities in Uganda.

Characteristics	Categories	Positive Urine Drug Test	Crude Odds Ratio (95% CI)	*p*-Value	Adjusted Odds Ratio (95%CI)*	*p*-Value
	All participants	55 (4.4)				
Gender	Male	50 (7.7)	1 (Reference)		1 (Reference)	
	Female	14 (2.4)	0.36 (0.16–0.81)	0.02	0.31 (0.13–0.74)	0.01
Age group	15-19	13 (2.7)	1 (Reference)		1 (Reference)	
	20-24	42 (5.5)	2.11 (1.34–3.34))	0.003	2.32 (1.42–3.78)	<0.001
Education	None/primary	52 (6.9)				
	Secondary and above	12 (2.5)	0.35 (0.19–0.64)	0.002	0.39 (0.21–0.7)	<0.001
Tribe	Central	19 (4.1)	1 (Reference)		1 (Reference)	
	Eastern	18 (4.4)	1.06(0.50–2.27)		1.00 (0.43–2.11)	
	Northern	1 (3.3)	0.81(0.09–7.61)		0.84 (0.09–7.71)	
	South-Western	12 (6.1)	1.53 (0.67–3.51)		1.35 (0.58–3.15)	
	Non-Ugandan	12 (10.5)	2.76 (1.42–5.37)		2.42 (1.18–5.00)	
	Other	2 (9.5)	2.46 (0.29–20.97)	0.14	2.06 (0.18–24.16)	0.28
Religion	Christian	48 (5.1)	1 (Reference)			
	Muslim	15 (5.2)	1.03 (0.62–1.71)		1.14 (0.68–1.92)	
	Traditional/other	1 (7.7)	1.55 (0.17–14.5)	0.91	0.87 (0.07–10.62)	0.87
Parity	0	31 (4.6)	1 (Reference)		1 (Reference)	
	1	23 (7.0)	1.54 (0.90–2.65)		2.07 (0.92–4.64)	
	≥2	10 (4.18)	0.90 (0.44–1.83)	0.13	1.58 (0.63–3.94)	0.21
Marital status	Cohabiting	5 (2.4)	1 (Reference)		1 (Reference)	
	Married	17 (4.3)	1.85 (0.83–4.11)		1.55 (0.71–3.38)	
	Separated/divorced/widowed	7 (8.4)	3.83 (1.15–12.80)		2.94 (0.80–10.81)	
	Single	35 (6.4)	2.86 (1.25–6.65)	0.11	3.69 (1.83-7.47)	0.01
Income	<200	20 (2.7)	1 (Reference)		1 (Reference)	
	200k-300k	19 (9.4)	3.7 (1.2–11.97)		2.37 (0.68–8.28)	
	300k-400k	8 (5.6)	2.2 (0.86–5.39)		1.11 (0.35-3.50)	
	≥400K	8 (5.8)	2.2 (1.12–4.528)	0.007	1.91 (0.75-4.91)	0.46
Occupation	Agriculture	9 (4.5)	1 (Reference)		1 (Reference)	
	Entertainment	6 (3.1)	0.68 (0.27–1.74)		0.91 (0.38–2.16)	
	Fishing related	39 (10.7)	2.53 (1.10–5.80)		1.51 (0.56–4.08)	
	Itinerary trade	5 (2.8)	0.60 (0.22–1.66)		0.72(0.25–2.12)	
	Unemployed	5 (1.9)	0.41 (0.14–1.21)	0.04	0.81(0.26–2.59)	0.69
	Other	0				
						
Duration in community	≤6 months	18 (3.4)	1 (Reference)			
	7 months to 1 year	17 (8.3)	2.59 (1.20–5.30)		2.12 (1.11–4.06)	
	1-4 years	20 (5.3)	1.61 (0.60–3.00)		1.12 (0.52–2.41)	
	≥ 5 years	9 (7.2)	2.22 (0.43–5.44)	0.08	1.35 (0.37–4.90)	0.10
						
Alcohol adverts	No	9 (3.6)	1 (Reference)		1 (Reference)	
	Yes	55 (5.6)	1.60 (0.87–2.95)	0.12	1.51 (.73–3.12)	0.25
						
School curriculum	No	19 (4.7)	1 (Reference)		1 (Reference)	
	Yes	45 (5.4)	1.15 (0.67–1.98)	0.59	1.64 (0.91–2.95)	0.09
						
Smoking	No	18 (1.7)	1 (Reference)		1 (Reference)	
	Yes	46 (23.1)	17.10 (8.32–35.02)	<0.001	13.05 (5.84–29.18)	<0.001
						
HIV	Negative	52 (4.4)	1 (Reference)		1 (Reference)	
	Positive	3 (4.8)	1.10 (0.27–4.26)	0.91	1.22 (0.19–8.01)	0.83
						
HSV2	Negative	40 (62.5)	1 (Reference)		1 (Reference)	
	positive	24 (37.5)	1.40 (0.84–2.24)	0.18	1.37 (0.81–2.30)	0.22
						
Depressive symptoms	Minimal	48 (4.3)	1 (Reference)		1 (Reference)	
	Mild	13 (11.2)	2.79 (0.93–8.34)		2.22 (0.83–5.91)	
	Moderate–severe	3 (17.7)	4.72 (1.42–15.68)	0.04	8.23 (1.82–37.16)	0.03

*All adjusted ORs controlled for age, gender, education, tribe and religion; adjusted ORs for parity, marital status, income, occupation, duration in community, alcohol adverts and school curriculum additionally controlled for each other; adjusted ORs for smoking, alcohol misuse, depressive symptoms, HIV and HSV2 additionally adjusted for each other (with the exception of HIV and HSV2, which were not controlled for each other).

## Supplementary Materials

The following are available online at https://www.mdpi.com/1660-4601/17/7/2401/s1, Table S1: Risk factors for alcohol misuse (PEth 16:0/18:1 ≥ 210 ng/mL) among young people aged 15–24 years in fishing communities in Uganda.
