# Supplementary material for: Epidemiology of Alcohol Misuse and Illicit Drug Use Among Young People Aged 15–24 Years in Fishing Communities in Uganda
Source: Int J Environ Res Public Health. 2020 Apr 1;17(7):2401. doi: 10.3390/ijerph17072401 (PMC7178227; doi:10.3390/ijerph17072401)
Supplement: Supplementary file 1 [file ijerph-17-02401-s001.pdf]

International Journal of
Environmental Research
and Public Health

MDPI

Supplamentary Material

Table 1. Risk factors for alcohol misuse (PEth 16:0/18:1 ≥210 ng/mL) among young people aged 15–24 years in fishing communities in Uganda.

Characteristic	Categories	Hazardous drinking (PEth 16:0/18:1 ≥210 ng/mL) N(%) *	Crude odds ratio (95% CI)	*P*-value	Adjusted odds ratio(95%CI)	*p*-value
	All participants	55 (4.3)				
Gender	Male	37 (5.5)	1 (Reference)		1 (Reference)	
	Female	18 (3.0)	0.52 (0.30–0.93)	0.03	0.55 (0.31–0.99)	0.05
Age	15-19	3 (0.6)	1 (Reference)		1 (Reference)	
	20-24	52 (6.6)	11.4 (3.54–36.65)	<0.001	12.67 (2.22–72.43)	0.007
Education	None/primary	45 (5.7)	1 (Reference)		1 (Reference)	
	Secondary and above	10 (2.0)	0.34 (0.18–0.65)	0.003	0.28 (0.14–0.54)	0.001
Tribe	Central	25 (5.3)	1 (Reference)		1 (Reference)	
	Eastern	12 (2.8)	0.52 (0.24–1.12)		0.47 (0.23–0.97)	
	Northern	-	-		-	
	South-Western	6 (3.0)	0.55 (0.22–1.40)		0.41 (0.16–1.06)	
	Non-Ugandan	12 (10.1)	2.02 (1.14–3.57)		1.72 (0.91–3.25)	
	Other	-	-	0.04	-	0.07
Religion	Christian	46 (4.8)	1 (Reference)		1 (Reference)	
	Muslim	8 (2.7)	0.55 (0.24–1.23)		0.57 (0.24–1.36)	
	Traditional/other	1 (7.2)	1.53 (0.17–13.48)	0.33	0.99 (0.08–12.95)	0.42
Parity	0	16 (2.3)	1 (Reference)		1 (Reference)	
	1	26 (7.5)	3.40 (1.24–9.34)		2.41 (0.79–7.38)	

	≥2	13 (5.3)	2.32 (0.82–6.56)	0.07	1.89 (0.47–7.63)	0.26
Marital status	Cohabiting	7 (3.1)	1 (Reference)		1 (Reference)	
	Married	20 (4.9)	1.59 (0.81–3.12)		1.24 (0.58–2.67)	
	Separated/divorced/ widowed	8 (9.1)	3.09 (1.09–8.78)		1.74(0.53–5.72)	
	Single	20 (3.4)	1.15 (0.57–2.30)	0.10	1.71 (0.80–3.71)	0.48
Income	<200	20 (2.6)	1 (Reference)		1 (Reference)	
	200k-300k	22 (10.4)	4.23 (2.25–8.31)		2.69 (1.27–5.72)	
	300k-400k	7 (4.7)	1.85 (0.74–4.66)		1.29 (0.42–3.99)	
	≥400K	6 (4.0)	1.57 (0.56–4.43)	0.002	0.88 (0.30–2.59)	0.03
Occupation	Agriculture	4 (2.0)	1 (Reference)		1 (Reference)	
	Entertainment	11 (5.6)	2.93 (1.04-8.21)		3.10 (1.13–8.51)	
	Fishing related	33 (8.8)	4.75 (1.62-13.91		3.68 (1.16–12.79)	
	Itinerary trade	4 (2.1)	1.08 (0.47-2.50)		1.12 (0.48–2.60)	
	Unemployed	2 (0.7)	0.36 (0.15-0.90)		0.70 (0.25–1.97)	
	Other	1 (2.6)	1.30 (0.14-12.43)	0.07	1.74 (0.28–10.72)	0.39
Duration in community	≤6 months	21 (3.8)	1 (Reference)		1 (Reference)	
	7 months to 1 year	9 (4.2)	1.11 (0.52-2.38)		1.01 (0.52–1.97)	
	1-4 years	17 (4.4)	1.15 (0.57-2.34)		0.75 (0.34–1.66)	
	≥5 years	8 (6.4)	1.71 (0.03-0.06)	0.79	0.96 (0.31–3.01)	0.88
Alcohol adverts	No	6 (2.3)	1 (Reference)		1 (Reference)	
	Yes	49 (4.8)	2.17 (0.86–5.57)	0.10	2.26 (0.92–5.55)	0.07
School curriculum	No	22 (5.2)	1 (Reference)		1 (Reference)	
	Yes	33 (3.9)	0.73 (0.36–1.50)	0.37	0.87 (0.42–1.82)	0.70
Smoking	No	25 (2.3)	1 (Reference)		1 (Reference)	
	Yes	30 (15.5)	7.08 (3.45–14.52)	<0.001	7.80 (2.67–22.8)	0.001

Illicit drug use	No	40 (3.4)	1 (Reference)		1 (Reference)	
	Yes	10 (15.6)	5.25 (2.15–12.78)	0.001	1.02 (0.29–3.60)	0.97
HIV	Negative	49 (4.1)	1 (Reference)		1 (Reference)	
	positive	6 (9.0)	2.32 (0.98–5.45)	0.06	1.29 (0.32–5.18)	0.71
HSV2	Negative	28 (3.3)	1 (Reference)		1 (Reference)	
	Positive	27 (6.7)	2.13 (1.20–3.79)	0.01	1.83 (0.88–3.84)	0.10
Depressive symptoms	Minimal	50 (4.5)	1 (Reference)		1 (Reference)	
	Mild	4 (3.2)	0.72 (0.21–2.47)		0.46 (0.09–2.31)	
	Moderate-severe	1 (4.6)	0.77 (0.19–3.06)	0.86	-	0.33

*Comparing PEth 16:0/18:1 ≥210 ng/mL to PEth 16:0/18:1 ≤ 20 ng/mL.
